# Disperse Dyes Based on Aminothiophenes: Their Dyeing Applications on Polyester Fabrics and Their Antimicrobial Activity

**DOI:** 10.3390/molecules18067081

**Published:** 2013-06-18

**Authors:** Saleh Mohammed Al-Mousawi, Morsy Ahmed El-Apasery, Huda M. Mahmoud

**Affiliations:** 1 Chemistry Department, Faculty of Science, Kuwait University, P.O. Box 5969, Safat 13060, Kuwait; E-Mail: elapaserym@yahoo.com; 2 Dyeing, Printing and Textile Auxiliaries Department, Textile Research Division, National Research Centre, Dokki, Giza 12622, Egypt; 3 Department of Biological Sciences, Faculty of Science, Kuwait University, P.O. Box 5969, Safat 13060, Kuwait; E-Mail: bsm8ham@yahoo.co.uk

**Keywords:** biological activity, polyester fabrics, disperse dyes, thienopyridazines

## Abstract

A series of monoazo disperse dyes derived from arylazothienopyridazines were synthesized. Fastness properties of dyed polyester samples were measured. Most of the dyed fabrics tested displayed excellent washing and perspiration fastness and moderate light fastness. Finally, the biological activity of the synthesized dyes against Gram positive bacteria, Gram negative bacteria and yeast were evaluated.

## 1. Introduction

Aminothiophene-based azo dyes are known as disperse dyes with excellent brightness of shade. This class of dyes was established as an alternative to more expensive anthraquinone dyes [1]. Thiophene-containing azo dyes have many advantages, including a colour deepening effect as an intrinsic property of the thiophene ring and small molecular structure leading to better dyeability. The heterocyclic nature of the thiophene ring has also allowed for excellent sublimation fastness on the dyed fibers [2]. A number of researchers have studied aminothiophene derivatives as azo disperse dyes in the dyeing of synthetic fibers [3,4,5,6,7] and blended polyester/wool fibers [8], This class of compounds also showed semiconducting properties and more recently, uses in optical data storage devices [9]. The synthesis of arylazothienopyridazines and their benzo-fused derivatives have been recently extensively studied [10,11]. In spite of a large number of reports on the utility of these compounds in the dye industry, to our knowledge, their corresponding arylazothienopyridazines have never been reported as potential monoazo disperse dyes. In continuation of our interest in the synthesis of arylazothieno-pyridazines and their condensed derivatives [12], this paper reports the synthesis of some arylazo-thienopyridazines using a conventional method and their application as disperse dyes on polyester fabrics. In addition the biological activity of the synthesized dyes against *Escherichia coli* and *Pseudomonas aeruginosa* (Gram negative bacteria), *Bacillus subtilis* and *Staphylococcus aureus* (Gram positive bacteria) and *Candida albicans* (yeast) was also evaluated.

## 2. Results and Discussion

### 2.1. Synthesis

Arylazopyridazinone **1** reacted with dimethylformamide dimethyl acetal (DMFDMA) in refluxing DMF for 6 hours affording dihydropyridazine-4-carbonitrile **2** that was readily converted into the pyrido[3,4-d]pyridazine-4,5-dione disperse dye **3** in 87% yield on treatment with ammonium acetate and acetic acid at reflux for 3 h.

**Scheme 1:**
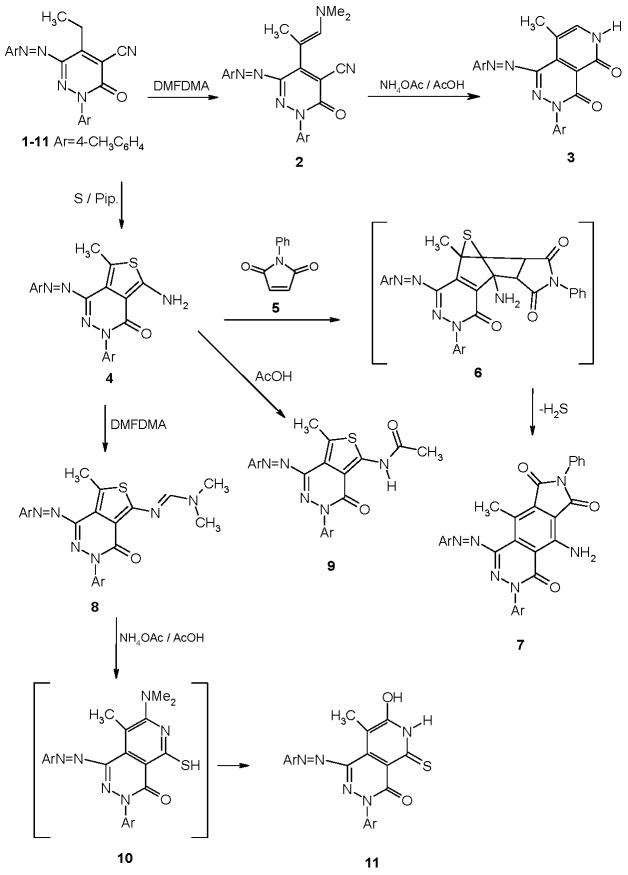
Preparation of monoazo disperse dyes.

**Figure 1:**
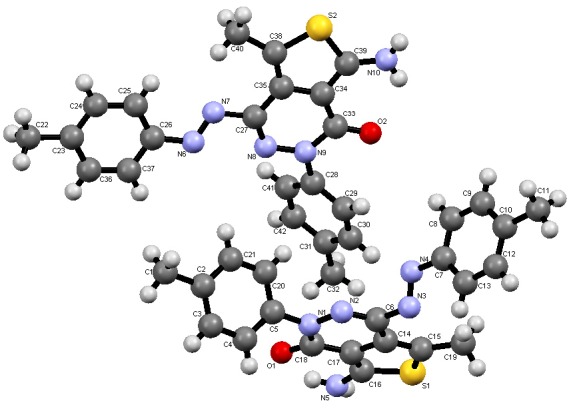
ORTEP plot of the X-ray crystallographic structure of **4**.

Compound **1** readily reacted with elemental sulphur in the presence of few drops of piperidine in dioxane as reaction medium to yield arylazoaminothienopyridazine disperse dye **4** in 89% yield. An X-ray crystal structure determination of **4** confirmed with certainly its structure [13] (*cf.* Scheme 1 and Figure 1). Typical to the established behavior of thienopyridazines, compound **4** reacted readily with *N*-phenylmaleimide in a mixture of acetic acid and dioxane to yield pyrrolo[3,4-g]phthalazine disperse dye **7** via the intermediary of the [4+2] cycloadduct **6**. 

**Figure 2:**
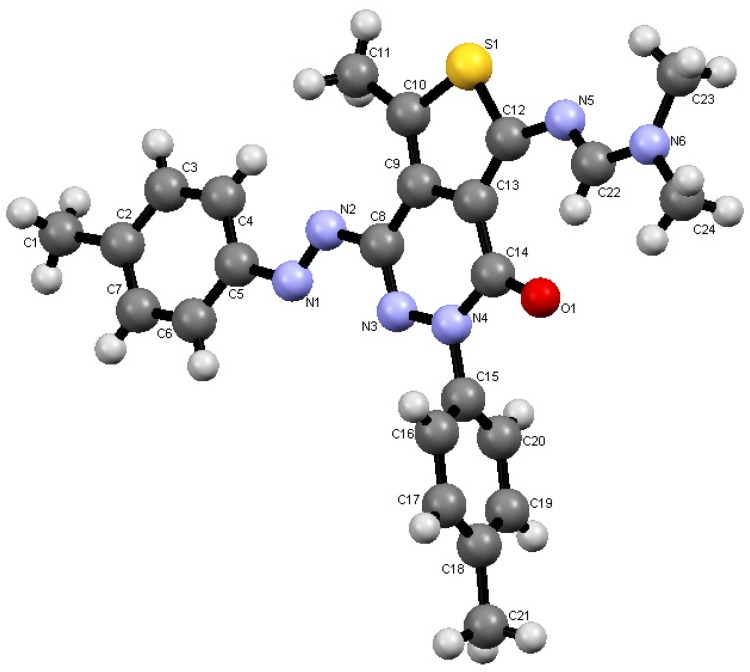
ORTEP plot of the X-ray crystallographic structure of **8**.

Reaction of compound **4** with DMFDMA afforded the corresponding amidine disperse dye **8**. The X-ray crystal structure determination of **8** confirmed with certainly its structure [13] (*cf.* Scheme 1 and Figure 2). Compound **8** upon heating with ammonium acetate in the presence of a few drops of acetic acid afforded the pyridopyridazine disperse dye **11** via the intermediacy of the [4+2] cycloadduct **10**. Hydrolysis of the dimethylamino moiety in the latter product afforded the final product **11**. Acylating **4** by refluxing in acetic acid resulted in the formation of the acetylamino disperse dye **9** in 80% yield.

### 2.2. Dyeing

Disperse dyes **3**, **4**, **7**–**9** and **11** were applied to polyester fabrics at 2% (dye shade), using the high temperature dyeing method (HT) at 130 °C. Yellowish-brown to brown color shades were obtained. The dyeing properties on the polyester fabrics were evaluated in terms of their fastness properties (e.g., fastnesses to washing, perspiration and light). The color of dyeing on polyester fabrics is expressed in terms of CIELAB values (Table 1), and the following CIELAB coordinates were measured: lightness (L*); chroma (C*); hue angle (*h*) from 0 to 360°; a*, whose value represents the degree of redness (positive) and greenness (negative); and b*, whose value represents the degree of yellowness (positive) and blueness (negative).

For dyeing polyester fabrics, in practical terms only disperse dyes are suitable. Through their hydrophobic properties, these dyes are capable of penetrating into the similarly hydrophobic polyester fibre. In general, the positive values of b* (yellow–blue axis) indicated that the color hues of the all disperse dyes on the polyester fabric shifted to the yellowish directions. The results listed in Table 1 show that the dyeing fabrics have different color strengths. The difference in color strength depends on the substitutes present and/or the position of the substitutes on the structure of the synthesized dyes. A chromophore-containing compound is called a “chromogen”: its color depends on the nature, number and position of the “auxochromes”. Auxochromes may shift the absorption towards higher wavelengths and thus the color of the dye will deepen. Such groups are called ’bathochromes’ (*i.e.*, having electron-donating substituents such as OH, NH_2_ and CH_3_); these are said to have a bathochromic effect. A group having the opposite, hyposochromic, effect is called a ‘hypochrome’ (*i.e.*, having electron-attracting substituents such as CHO, COR and NO_2_). The results given in Table 1, show that the magnitude of color strength *K/S* of dye **4** (17.78) at *λ*_max_ 464 is much higher than the color of dyes **8** and **9** (10.49 and 16.30) at *λ*_max_ 448 and 431, respectively. The results listed in Table 1, show also that the heteroaromatic diazo component can provide disperse dyes with bathochromic shifts due to the stabilization of the excited state.

**Table 1:** Shade and optical measurements of the azo disperse dyes on the polyester fabrics.

Dye No.	Color on polyester (2% shade)	L*	a*	b*	C*	h*	*K/S*
**3**	Yellowish-brown	50.77	4.40	20.70	21.17	78.01	13.33
**4**	Brown	46.06	24.84	29.03	38.21	49.45	17.78
**7**	Cream	83.98	11.31	42.49	43.97	75.09	6.79
**8**	Light brown	68.84	22.97	39.59	45.77	59.88	10.49
**9**	Brownish-yellow	66.03	22.50	60.44	64.49	69.58	16.30
**11**	Light cream	82.96	9.55	33.54	34.87	74.11	6.76

The physical data for the dyed fabrics given in Table 2, shows that the disperse dyeing displayed moderate fastness levels to light. The light fastness of each of the dyes was measured by employing the standard method for determination of color fastness of textiles. Several reports [14] suggest that fading of azo dyes is mainly a consequence of decomposition of the –N=N– moiety either by oxidation, reduction or photolysis. The rates of these processes should be sensitive to the chemical structure of the dye, the type of substrate and treatment conditions. Since the dyed substrate employed in this study is a polyester fabrics (*i.e.*, non-proteinic), the fading process likely occurs by oxidation [15]. The ease of oxidation of azo linkages should be a function of electron density. Therefore, electron donating substituents on this moiety should increase the fading rate while electron withdrawing groups should decrease the rate. This proposal is in agreement with the observed results (Table 2) which demonstrate that the presence of a hydroxyl group in dye **11** causes decrease of light fastness to 3. On the other hand, high light fastness of dye **7** to 6, is may be attributed to hydrogen bonding between the amino group and carbonyl group cause aggregation of this dye sequent, the fading is decreased [16]. 

In addition the results obtained showed that dyed fabrics have excellent fastness levels to washing and perspiration fastness properties that may be due to the absence of solubilizing groups, which affects solubility, and wash ability of the dye-out of the fabrics or to the size of the dye molecule is considered relatively big.

**Table 2:** Fastness properties of monoazo disperse dyes on polyester fabrics.

Dye No.	Wash fastness			Perspiration fastness						Light fastness
				Alkaline			Acidic			
	Alt	SC	SW	Alt	SC	SW	Alt	SC	SW	
**3**	5	5	5	5	5	5	5	5	5	2
**4**	5	5	5	5	4–5	5	5	5	5	2
**7**	5	5	5	5	5	5	5	5	5	6
**8**	5	4–5	5	5	4–5	5	5	4–5	5	3
**9**	5	5	5	5	5	5	5	5	5	3
**11**	5	5	5	5	5	5	5	5	5	3

Alt = alteration; SC = staining on cotton; SW = staining on wool.

### 2.3. Antimicrobial Activities

The antimicrobial activities of the synthesized disperse dyes were screened against selected bacteria and fungi by the agar well diffusion method and their inhibition zones diameters, given in Table 3, reveal that the tested dyes **3** and **9** showed positive antimicrobial activities against at four of the tested microorganisms. Dye **3** showed strong activities (>10 mm inhibition zone) against *E. coli*, whereas dyes **7**, **8** and **11** showed no activities against any of the tested microorganisms used in the study. Only dye **9** showed a significant inhibition zone <10 mm against *Candida albicans*.

**Table 3:** Diameter of the zones of inhibition of the tested disperse dyes against Gram positive, Gram negative bacteria and yeast.

Dye No.	Inhibition zone diameter				
	*G+ve* bacteria		*G-ve* bacteria		Fungi
	*B. subtilis* Mean ± SD	*S. aureus* Mean ± SD	*E. coli* Mean ± SD	*P. aeruginosa* Mean ± SD	*C. albicans* Mean ± SD
**3**	8.4 ± 0.7	6.6 ± 0.2	11.2 ± 0.2	8.6 ± 1.3	-
**7**	-	-	-	-	-
**8**	-	-	-	-	-
**9**	5.1 ± 0.5	5.1 ± 0.2	7.4 ± 0.8	6 ± 0.3	6.4 ± 0.4
**11**	-	-	-	-	-
Ampicillin *	10 ± 0.5	3.5 ± 0.2	10.8 ± 0.7	9.5 ± 2.6	
Cycloheximide **					-

(-) no inhibition; * Ampicillin: Antibacterial (100 mg·mL^−1^); ** Cycloheximide: Antifungal (100 mg·mL^−1^); SD = Standard Deviation.

**Figure 3:**
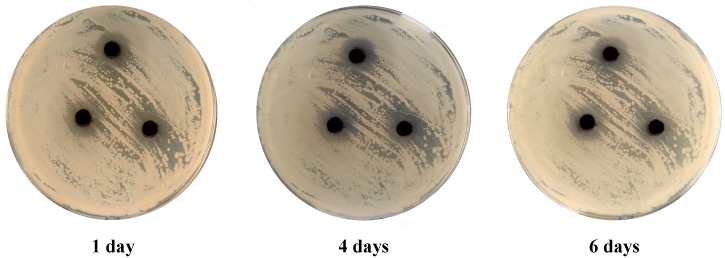
*E. coli*. treated with 100 mg/mL of dye **3** after 1, 4, and 6 days of incubation.

**Figure 4:**
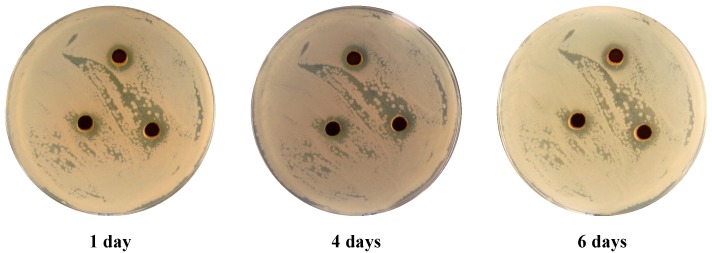
*E. coli*. treated with 100 mg/mL of dye **9** after 1, 4, and 6 days of incubation.

In addition, disperse dyes **3** and **9** (Figure 3, Figure 4) showed cytolytic effect even after six days of incubation, there were no growth recorded in the inhibited zone for all five tested microbes. 

Currently, we are inspecting the biological activity of the polyester fabrics dyed with aminothiophene dyes against Gram positive bacteria, Gram negative bacteria and yeast.

## 3. Experimental

### 3.1. General

Melting points were recorded on a Gallenkamp apparatus. IR spectra were recorded using KBr pellets on a JASCO FTIR-6300 FT-IR spectrophotometer. ^1^H- and ^13^C-NMR spectra were recorded on Bruker DPX 400 MHz or AvanceII 600 MHz super-conducting NMR spectrometers with proton spectra measured at 400, 600 MHz and carbon spectra at 100 and 150 MHz, respectively. Mass spectra were measured on a high resolution GC/MS DFS-Thermo instrument. Microanalyses were performed on an Elementar-Vario Microcube analyzer. The crystal structure of compounds **4** and **8** were determined by Bruker AXS X8 Prospector Single Crystal X-Ray Diffractometer at Kuwait University. The dyeing of polyester fabrics were conducted using LINITEST+Laboratory High Temperature Dyeing and Fastness System (ATLAS, Lichtenstein, Germany). The colorimetric parameters of the dyed polyester fabrics were determined on a reflectance spectrophotometer (UltraScan PRO D65, HunterLab, Virginia, VA, USA).

*6-p-Tolyldiazenyl)-5-(1-(dimethylamino)prop-1-en-2-yl)-3-oxo-2-p-tolyl-2,3-dihydropyridazine-4 carbo-nitrile* (**2**). A solution of compound **1** (3.55 g, 10 mmol) and DMFDMA (1.19 g, 10 mmol) was refluxed for 2 h then allowed to cool to room temperature. The solid product obtained was crystallized from dioxane to afford compound **2** as a yellow powder (73%), m.p. 292–294 °C; IR (KBr): 2237 (CN), 1655 (CO) cm^-1^; ^1^H-NMR (DMSO-d_6_): δ = 2.08 (s, 3H, CH_3_), 2.23 (s, 6H, 2(*p*-tolyl-CH_3_)), 2.27 (s, 6H, N-(CH_3_)_2_), 6.80 (d, 2H, *J* = 7.7 Hz, *p*-tolyl-H), 7.01 (d, 2H, *J* = 6.6 Hz, *p*-tolyl-H), 7.07–7.13 (m, 2H, *p*-tolyl-H), 7.21 (d, 2H, *J* = 8.1 Hz, *p*-tolyl-H), 7.32 (d, 1H, *J* = 7.9 Hz, vinylic-H). MS, *m/z* (%), 412 ([M]^+^, 45), UV/Vis at *λ*_max_(DMF) = 432 nm*.* Anal. Calcd. For C_24_H_24_N_6_O: C, 69.88; H, 5.86; N, 20.37. Found C, 69.91; H, 5.44; N, 19.73. 

*8-Methyl-3-p-tolyl-1-(p-tolyldiazenyl)pyrido[3,4-d]pyridazine-4,5(3H,6H)-dione*(**3**). A solution of compound **2** (4.32 g, 10 mmol), acetic acid (15 mL) and ammonium acetate (1.15 g, 15 mmol) was refluxed for 3 h, then allowed to cool to room temperature. The solid product so formed was collected by filtration and crystallized from acetic acid. Compound **3** was obtained as a light brown powder (87%), m.p. 226–228 °C; IR (KBr): 3451 (NH), 1706, 1659 (CO) cm^-1^; ^1^H-NMR (DMSO-d_6_): δ = 2.16 (s, 3H, CH_3_), 2.31 (s, 6H, 2(*p*-tolyl-CH_3_)), 7.10–7.40 (m, 4H, *p*-tolyl-H), 7.46 (d, 2H, *J* = 8.4 Hz, *p*-tolyl-H), 7.52 (d, 2H, *J* = 7.2 Hz, *p*-tolyl-H), 8.79 (s, 1H, pyridyl-H). 9.91 (s, 1H, NH).MS, *m/z* (%), 383 ([M-2]^+^, 32), UV/Vis at *λ*_max_(DMF) = 590. Anal. Calcd. For C_22_H_19_N_5_O_2_: C, 68.56; H, 4.97; N, 18.17. Found C, 68.58; H, 4.99; N, 18.26. 

*7-Amino-5-methyl-2-p-tolyl-4-p-tolylazo-2H-thieno[3,4-d]pyridazin-1-one* (**4**). A mixture of compound **1** (3.55 g, 10 mmol) and elemental sulphur (0.32 g, 10 mmol) and a few drops of piperidine in dioxane (10 mL) was refluxed for 4 h then allowed to cool to room temperature. The crude product was poured onto water, the solid product, so formed, was collected by filtration and crystallized from ethanol. Compound **4** was obtained as wine red crystals (89%), m.p. 209 °C (Lit. [12] m.p. 208–209 °C), IR (KBr): 3321, 3278 (NH_2_), 1642 (CO) cm^-1^; ^1^H-NMR (DMSO-d_6_): δ = 2.30 (s, 3H, CH_3_), 2.37 (s, 3H, *p*-tolyl-CH_3_), 2.40 (s, 3H, *p*-tolyl-CH_3_), 7.21 (d, 2H, *J* = 10.2 Hz, *p*-tolyl-H), 7.32 (d, 2H, *J* = 9.6 Hz, *p*-tolyl-H), 7.38 (s, 2H, NH_2_), 7.39 (d, 2H, *J* = 7.2 Hz, *p*-tolyl-H), 7.78 (d, 2H, *J* = 7.2 Hz, *p*-tolyl-H). ^13^C NMR (DMSO-d_6_): δ = 160.77 (CO), 159.2, 150.4, 150.1, 144.0, 139.5, 136.9, 130.7, 129.5, 126.3, 123.6, 121.9. 116.7, 104.7, 21.5 (*p*-tolyl-CH_3_), 21.0 (*p*-tolyl-CH_3_), 14.7 (CH_3_). MS (EI) *m/z =* 389 (M]^+^, 100); UV/Vis at *λ*_max_(DMF) = 464 nm. Anal. Calcd for C_21_H_19_N_5_OS: C, 64.76; H, 4.92; N, 17.98; S, 8.23. Found: C, 65.09; H, 5.08; N, 17.90; S, 8.21.

*9-Amino-5-methyl-7-phenyl-2-p-tolyl-4-p-tolylazo-2H-pyrrolo[3,4-g]phthalazine-1,6,8-trione* (**7**). A solution of compound **4** (3.89 g, 10 mmol), N-phenylmaleimide (1.73 g, 10 mmol) and a few drops of acetic acid was refluxed in dioxane (15 mL) for 10 h. The solid products, so formed, were collected by filtration and crystallized from DMF. Compound **7** was obtained as orange crystals (79%), m.p. 302–304 °C) (Lit. [12] m.p. 300–302 °C), IR (KBr): 3435, 3310 (NH_2_),1752, 1704, 1645 (CO) cm^−1^; ^1^H-NMR (DMSO-d_6_): δ = 2.42 (s, 3H, CH_3_), 2.48 (s, 3H, *p*-tolyl-CH_3_), 2.75 (s, 3H, *p*-tolyl-CH_3_), 7.36 (d, 2H, *J* = 7.8 Hz, *p*-tolyl-H), 7.50 (d, 4H, *J* = 8.1 Hz, *p*-tolyl-H), 7.57–7.64 (m, 5H, phenyl-H), 7.94 (d, 2H, *J* = 7.7 Hz, *p*-tolyl-H), 9.47 (s, 2H, NH_2_). MS (EI) *m/z =* 528 ([M]^+^, UV/Vis at *λ*_max_(DMF) = 471 nm. Anal. Calcd for C_31_H_24_N_6_6O_3_: C, 70.44; H, 4.58; N, 15.90. Found: C, 69.99; H, 4.58; N, 15.96.

*N,N-Dimethyl-N'-(7-methyl-4-oxo-3-p-tolyl-1-p-tolylazo-3,4-dihydrothieno[3,4-d]pyridazin-5-yl)-formamidine* (**8**). A mixture of compound **4** (3.89 g, 10 mmol) and DMFDMA (1.19 g, 10 mmol) in dimethylformamide (15 mL), was refluxed for 1 h. The solvent was evaporated and then the residue was washed with ethanol. The solid product, so formed, was collected by filtration and crystallized from dimethylformamide. Compound **8** was obtained as wine red crystals (93%), m.p. 307 °C (Lit. [12] m.p. 203–205 °C), IR (KBr): 1651 (CO) cm^-1^. ^1^H-NMR (DMSO-d_6_): δ = 2.31 (s, 3H, CH_3_), 2.39 (s, 3H, *p*-tolyl-CH_3_), 2.53 (s, 3H, *p*-tolyl-CH_3_), 2.96 (s, 3H, N-CH_3_), 3.04 (s, 3H, N-CH_3_), 7.22 (d, 2H, *J* = 9.0 Hz, *p*-tolyl-H), 7.30 (d, 2H, *J* = 9.0 Hz, *p*-tolyl-H), 7.41 (d, 2H, *J* = 7.8 Hz, *p*-tolyl-H), 7.80 (d, 2H, *J* = 8.4 Hz, *p*-tolyl-H), 7.92 (s, 1H, amidine-H). ^13^C NMR (DMSO-d_6_): δ = 163.7 (CO), 157.5, 157.3, 150.5, 149.9, 144.0, 139.2, 137.1, 130.7, 129.3, 126.8, 125.4, 123.8, 123.7, 112.9, 34.7 (N-CH_3_), 21.5 (*p*-tolyl-CH_3_), 21.0 (*p*-tolyl-CH_3_), 15.8 (CH_3_). MS (EI) *m/z =* 444 (M]^+^, 100), UV/Vis at *λ*_max_(DMF) = 448 nm. Anal. Calcd for C_24_H_24_N_6_OS: C, 64.84; H, 5.44; N, 18.90; S, 7.21. Found: C, 64.58; H, 5.59; N, 18.70; S, 7.16.

*N-(7-Methyl-4-oxo-3-p-tolyl-1-p-tolylazo-3,4-dihydrothieno[3,4-d]pyridazin-5-yl)acetamide* (**9**). A solution of compound **4** (3.89 g, 10 mmol) in acetic acid (20 mL) was refluxed for 3h. The solvent was evaporated and then the residue was washed with ethanol. The solid product, so formed, was collected by filtration and crystallized from dioxane. Compound **9** was obtained as brown crystals (80%), m.p. 209 °C) (Lit. [12] m.p. 207–209 °C), IR (KBr): 3270 (NH), 1673, 1641 (CO) cm^-1^. ^1^H-NMR (DMSO-d_6_): δ = 2.30 (s, 3H,COCH_3_), 2.26 (s, 3H, *p*-tolyl-CH_3_), 2.33 (s, 3H, *p*-tolyl-CH_3_), 2.39 (s, 3H, CH_3_), 7.27 (d, 2H, *J* = 9.0 Hz, *p*-tolyl-H), 7.40–7.44 (m, 4H, *p*-tolyl-H), 7.83 (d, 2H, *J* = 7.8 Hz, *p*-tolyl-H), 11.03 (s, 1H, NH). ^13^C NMR (DMSO-d_6_): δ = 167.6 (CO), 158.3, 153.5, 149.9, 144.4, 142.7, 138.3, 137.4, 130.8, 129.5, 126.6, 123.8, 120.8, 119.8, 112.4, 23.1 (*p*-tolyl-CH_3_), 21.1 (*p*-tolyl-CH_3_), 16.5 (CH_3_), 14.8 (CH_3_). MS (EI) *m/z =* 431 (M]^+^, 100), UV/Vis at *λ*_max_(DMF) = 431 nm. Anal. Calcd for C_23_H_21_N_5_O_2_S: C, 64.02; H, 4.91; N, 16.23; S, 7.43. Found: C, 63.88; H, 4.89; N, 16.36; S, 7.34.

*7-Hydroxy-8-methyl-5-thioxo-3-p-tolyl-1-p-tolylazo-5,6-dihydro-3H-pyrido[3,4-d]pyridazin-4-one* (**11**). A mixture of compound **8** (4.44 g, 10 mmol) acetic acid (10 mL) and ammonium acetate (3.45 g, 30 mmol) was refluxed for 3 h. The crude product was poured onto water, the solid product, so formed, was collected by filtration and crystallized from dioxane/ethanol (3:1). Compound **11** was obtained as a light violet powder (68%), m.p. 297 °C (Lit. [12] m.p. 295–297 °C), IR (KBr): 3440 (OH), 3275 (NH), 1681, 1641 (CO) cm^-1^. ^1^H-NMR (DMSO-d_6_): δ = 2.37 (s, 3H, CH_3_), 2.43 (s, 3H, *p*-tolyl-CH_3_), 2.66 (s, 3H, *p*-tolyl-CH_3_), 7.29 (d, 2H, *J* = 7.8 Hz, *p*-tolyl-H), 7.44 (t, 4H, *J* = 7.4 Hz, *p*-tolyl-H), 7.87 (d, 2H, *J* = 7.8 Hz, *p*-tolyl-H), 8.61 (s, 1H, NH), 11.7 (s, 1H, OH). ^13^C NMR (DMSO-d_6_): δ = 157.8, 151.3, 151.0, 144.4, 141.1, 138.4, 138.2, 130.6, 130.2, 130.0, 129.6, 126.4, 125.6, 124.5, 120.2, 22.3 (*p*-tolyl-CH_3_), 21.8 (*p*-tolyl-CH_3_), 15.7 (CH_3_). MS (EI) *m/z =* 417 (M]^+^, 100), Anal. Calcd for C_22_H1_19_N_5_O_2_S: C, 63.29; H, 4.59; N, 16.78; S, 7.68. Found: C, 63.26; H, 4.78; N, 16.75; S, 7.56.

### 3.2. High Temperature Dyeing Method (HT)

#### 3.2.1. Materials

Polyester 100% (150 130 g/m^2^, 70/2 denier) was used. The fabric was treated before dyeing with a solution containing non-ionic detergent (Sera Wash M-RK, 5 g/L) and sodium carbonate (2 g/L) in a ratio of 50:1 at 60 °C for 30 min, then thoroughly washed with water and air dried at room temperature.

#### 3.2.2. Dyeing

The dye baths were prepared from the dye (2% weight of fibre) to a final liquor of 50:1, w/w. The pH value of the bath was adjusted to 4.5–5 with acetic acid (10%) in the presence of a 1:1 ratio of the dispersing agent (Sera Gal P-LP). The temperature was raised to 130 °C at the rate of 7 °C/min, and dyeing continued for 60 min. After dyeing, the fabrics were thoroughly washed and then subjected to a surface reduction clearing [(2 g NaOH + 2 g sodium hydrosulphite)/L]. The samples were heated in this solution for 30 min at 80 °C and then thoroughly washed and air-dried. 

### 3.3. Color Measurements and Analyses

#### 3.3.1. Color Measurements

The colorimetric parameters (Table 1) of the dyed polyester fabrics were determined on a reflectance spectrophotometer. The color yields of the dyed samples were determined by using the light reflectance technique performed on (UltraScan PRO D65) UV/VIS Spectrophotometer. The color strengths, expressed as *K/S* values, were determined by applying the Kubelka-Mink equation as follows:

K/S = [(1 − R)^2^/2R] − [(1 − R_o_)^2^/2R_o_]

where *R* = decimal fraction of the reflectance of the dyed fabric; *R_o_* = decimal fraction of the reflectance of the undyed fabric; *K* = absorption coefficient; *S* = scattering coefficient.

#### 3.3.2. Fastness Testing

##### 3.3.2.1. Fastness to Washing

After washing using 2 g/L of the nonionic detergent Hostapal CV at 80 °C for 15 min, the dyed fabrics were tested by using standard methods [17]. A specimen of dyed polyester fabric was stitched between two pieces of undyed cotton and wool fabrics, all of equal length, and then washed at 95 °C for 30 min. The staining on the undyed adjacent fabrics was assessed according to the following gray scale: 1—poor, 2—fair, 3—moderate, 4—good, 5—excellent.

##### 3.3.2.2. Fastness to Perspiration

The samples were prepared by stitching a piece of dyed polyester fabric between two pieces of cotton and wool fabrics, all of equal length, and then immersed in the acid or alkaline solution for 30 min. The staining on the undyed adjacent fabrics was assessed according to the following gray scale: 1—poor, 2—fair, 3—moderate, 4—good, 5—excellent. The acid solution (pH = 4.5) contains sodium chloride (10 g/L), sodium dihydrogen orthophosphate (1 g/L) and histidine monohydrochloride (0.25 g/L). The alkaline solution (pH = 9.5) contains sodium chloride (10 g/L), disodium orthophosphate (1 g/L) and histidine monohydrochloride (0.25 g/L).

##### 3.3.2.3. Fastness to Light

Light fastness was determined by exposing the dyed polyester fabrics on a Xenotest 150 (Original Hanau, Lichtenstein, Germany). Chamber temperature: 25–30 °C, black panel temperature: 60 °C, relative humidity: 50%–60%, dark glass UV filter system) for 40 h. The changes in color were assessed according to the following blue scale: 1—poor, 3—moderate, 4—good, 6—very good, 8—excellent.

### 3.4. Antimicrobial Activities Test

The antimicrobial activities of different arylazothienopyridazines disperse dyes were tested using Agar-well diffusion technique [18] against five different microbial cultures. Pure cultures of *Escherichia coli* and *pseudomonas aeruginosa* (Gram negative bacteria), *Bacillus subtilis* and *Staphylococcus aureus* (Gram positive bacteria) and *Candida albicans* (yeast) were used in the test. An aliquot of 0.1 mL of each bacterial strain was inoculated and spread on nutrient agar (NA) while 0.1 mL of the yeast was spread on potato dextrose agar (PDA). The inoculated plates were supplied with 100 µL of each of the tested dyes with a total final concentration of 10 mg mL^−1^. The dyes were included in 4 mm wells produced by sterile cork borer. The NA plates were incubated at 37 °C for 24 h while PDA plates were incubated at 25 °C for 24–48 h. The zones of inhibition around the wells were determined and the average based on three replica was recorded. Cycloheximide and ampicillin were both used as references in the experiment, where cycloheximide known to inhibit eukaryotic organisms while ampicillin inhibits prokaryote organisms. All plates were kept for six days after inoculation and the changes in the inhibition zone was monitored and documented by photography in order to determine on the cytolytic and cytostatic effect of the tested disperse dyes.

## 4. Conclusions

In summary, a series of monoazo disperse dyes based on arylazothienopyridazines were synthesized. The dyes produced in this manner were then applied to polyester fabrics by using a high temperature dyeing method at 130 °C. The dyed polyester fabrics, which display yellowish-brown to brown hues, displayed excellent washing and perspiration fastness and moderate light fastness. Finally, the biological activity of the synthesized compounds against Gram positive bacteria, Gram negative bacteria and yeast was tested. 
